# RocSampler: regularizing overlapping protein complexes in protein-protein interaction networks

**DOI:** 10.1186/s12859-017-1920-5

**Published:** 2017-12-06

**Authors:** Osamu Maruyama, Yuki Kuwahara

**Affiliations:** 1 Institute of Mathematics for Industry, Kyushu University, 744 Motooka, Nishi-ku, Fukuoka, 819-0395 Japan; 2 Graduate School of Mathematics, Kyushu University, 744 Motooka, Nishi-ku, Fukuoka, 819-0395 Japan

**Keywords:** Protein-protein interaction, Protein complex, Markov chain Monte Carlo, RocSampler, Regularization term

## Abstract

**Background:**

In recent years, protein-protein interaction (PPI) networks have been well recognized as important resources to elucidate various biological processes and cellular mechanisms. In this paper, we address the problem of predicting protein complexes from a PPI network. This problem has two difficulties. One is related to small complexes, which contains two or three components. It is relatively difficult to identify them due to their simpler internal structure, but unfortunately complexes of such sizes are dominant in major protein complex databases, such as CYC2008. Another difficulty is how to model overlaps between predicted complexes, that is, how to evaluate different predicted complexes sharing common proteins because CYC2008 and other databases include such protein complexes. Thus, it is critical how to model overlaps between predicted complexes to identify them simultaneously.

**Results:**

In this paper, we propose a sampling-based protein complex prediction method, RocSampler (Regularizing Overlapping Complexes), which exploits, as part of the whole scoring function, a regularization term for the overlaps of predicted complexes and that for the distribution of sizes of predicted complexes. We have implemented RocSampler in MATLAB and its executable file for Windows is available at the site, http://imi.kyushu-u.ac.jp/~om/software/RocSampler/.

**Conclusions:**

We have applied RocSampler to five yeast PPI networks and shown that it is superior to other existing methods. This implies that the design of scoring functions including regularization terms is an effective approach for protein complex prediction.

## Background

In recent years, protein-protein interaction (PPI) datasets have been recognized as important resources to elucidate various biological processes and cellular mechanisms. The prediction of protein complexes from PPIs (see, for example, survey papers [1–3]) is one of the most challenging inference problems from PPIs because protein complexes are essential entities in the cell. Proteins’ functions are manifested in the form of a protein complex. Thus, the identification of protein complexes is necessary for the precise description of biological systems.

For protein complex prediction, many computational methods have been proposed, which were directly or indirectly designed based on the observation that densely connected subgraphs, or clusters of proteins, of a whole PPI network often overlap with known complexes. This observation is often valid for relatively large protein complexes. However, small complexes, consisting of two or three proteins, form a major category of the known complexes of an organism [4, 5]. For example, a yeast protein complex database, CYC2008 [6], with 408 protein complexes includes 172 (42%) complexes consisting of two different proteins (called heterodimeric complexes), and 87 (21%) complexes consisting of three different proteins (called heterotrimeric complexes). Unfortunately, the density measure for a cluster of proteins, being a predicted complex, works less for smaller ones because the connectivity of PPIs within such a complex has small variations. For example, a cluster with two components either has an interaction or not. Thus, how to predict small complexes accurately is a critical issue,

To resolve this issue, we have proposed a sampling-based method for predicting protein complexes, PPSampler2 [4]. The concept of PPSampler2 involves regulating the frequency of the sizes of predicted clusters by a regularization term designed based on the observation that the distribution of the sizes of the complexes of an organism (see, for example, CYC2008 [6] for yeast and CORUM [7] for human) can be approximated by a power-law distribution. Namely, the regularization term evaluates how the distribution of the sizes of predicted clusters is likely to be a power-law distribution. The regularization term is used as part of the whole scoring function of PPSampler2. As a result, it is possible to identify small predicted complexes with relatively high accuracy.

However, there is a drawback to the model for the collection of clusters of proteins predicted by PPSampler2. This model involves a *partition* of all proteins in a given PPI network, and every element with two or more proteins is taken as a predicted complex. Thus, any two predicted complexes are exclusive, namely, they never share any common proteins due to the structure of partition. This partition model is also adopted by the Markov cluster algorithm (MCL), which is a popular node-clustering algorithm for an edge-weighted undirected graph based on the simulation of stochastic flow in the graph [8]. On the other hand, it is known that many complexes overlap with each other, namely they share common proteins. Actually, CYC2008 has 216 pairs of complexes sharing one or more common proteins. In this sense, the partition model is not the best model for a collection of predicted complexes. However, PPSampler2 and MCL are reported to achieve relatively good performance [4]. This implies that the partition model is a good approximation model for a set of predicted complexes.

Some existing methods *indirectly* allow predicted complexes to overlap with each other. Such methods often adopt the same scheme, which can be called the *cluster-expansion* approach. This involves repeatedly expanding a cluster of proteins by adding a protein out of the cluster, where an initial cluster is a cluster with either a single protein or a pair of proteins sharing an interaction, until a stop criterion is satisfied. After this expansion process is applied to all initial clusters, some of the resulting clusters can overlap with each other. If two predicted clusters have a large overlap, the high-scoring one remains and the other is discarded, or they are merged into one. This pruning process is repeated until there are no large overlaps between clusters. As a result, some clusters still overlap with each other. Examples of the cluster-expansion approach are ClusterONE [9], RRW [10], and NWE [11].

In this work, to address both of the issues of predicting small complexes and overlapping complexes simultaneously, we improve PPSampler2 by relaxing the partition model for a set of predicted complexes, so that predicted complexes are allowed to overlap with each other. To realize this relaxation, we propose a regularization term for controlling overlaps of predicted complexes, and add it as part of the whole scoring function of the new method. Furthermore, we have designed a proposal function, by which a current set of predicted complexes, some of which can overlap with each other, is partially modified into a new one. We call the resulting method RocSampler (Regularizing Overlapping Complexes). In addition, RocSampler uses refined terms of the scoring function of PPSampler2. We have empirically shown that RocSampler is superior to existing methods on five different yeast PPI datasets.

## Methods

We formulate a scoring function, *f*(*X*,*γ*), where *X* is a set of predicted clusters of proteins, which are allowed to overlap with each other, and *γ* is a scaling exponent of a power-law for the frequency of the size of predicted clusters in *X*. The probability, *P*(*X*,*γ*), of (*X*,*γ*) is given by

$$ P(X,\gamma) \propto \exp\left(-\frac{f(X,\gamma)}{T} \right) $$

where *T* is a positive real number, called a temperature parameter. Note that the lower *f*(*X*,*γ*) is, the higher *P*(*X*,*γ*) is.

We construct a Metropolis-Hastings algorithm for *P*(*X*,*γ*) with a fixed constant, *T*. This algorithm generates a sequence of samples from the distribution over (*X*,*γ*). Furthermore, for the Metropolis-Hastings algorithm, we introduce a cooling scheme, that is, a way of decreasing *T* gradually. Thus, the resulting method becomes a simulated annealing algorithm, shown in Algorithm 1, where a state of (*X*,*γ*) is denoted by *Z* for simplicity. We call the resulting algorithm RocSampler (Regularizing Overlapping Complexes). Among all samples, the one whose score is lowest is returned as the output of an execution.



In the subsequent section, we give the models of the input and output of the scoring function, *f*(*X*,*γ*), and some notations used throughout this paper. After that, we describe three key components of our methods: (i) the scoring function, *f*(*X*,*γ*), (ii) a proposal function that randomly generates a candidate state, (*X* ^′^,*γ* ^′^), from a current one, (*X*,*γ*), and (iii) a cooling scheme of *T*.

### Notations

A PPI network is represented as an undirected, edge-weighted graph, *G*=(*V*,*E*,*w*), where a node in *V* represents a protein, an edge in *E* is a PPI, and $w:E \to \mathcal {R}$ is a mapping from an edge in *E* to a weight in the interval, [0,1]. Additionally, we suppose that, for *e*={*u*,*v*}∉*E*, *w*(*e*)=0. We suppose that any self-loops, {*u*,*u*} where *u*∈*V*, are not included in *E*. If self-loops are included in a given data set, they are removed in a preprocessing step. For a subset, *x*, of *V*, we define *w*(*x*) as the sum of the weights of the interactions included in *x*, that is,

$$ w(x) = \sum_{u, v \in x} w(u,v). $$

Furthermore, for *u*∈*V* and *x*⊆*V*, we denote by *w*(*u*,*x*) the sum of weights of interactions between *u* and proteins in *x*, that is,

$$ w(u,x) = \sum_{v \in x} w(u,v). $$

We will use this notation in two different contexts, one of which is the case where *u* is outside of *x* and the other in which it is not.

We consider a subset of *V* as a predicted complex, and call it a predicted *cluster* to clearly distinguish it from a known complex. We denote a set of predicted clusters by

$$ X = \left\{ x_{1}, x_{2}, \ldots, x_{n} \subseteq V | |x_{i}| \ge 2 \right\}. $$

Every predicted cluster, *x*_*i*_∈*X*, should have two or more components as it models a protein complex. Note that, in this model, clusters are allowed to overlap with each other.

The Jaccard index between subsets of *V*, *x* and *x* ^′^, which is defined as

$$ J(x,x') = \frac{|x \cap x'| }{ |x \cup x'| }, $$

is often used as a similarity measure between two sets. We use this measure in determining whether or not a predicted cluster, *x*, and a known complex, *x* ^′^, match with each other, and in evaluating dissimilarity between *x* and *x* ^′^, which is explained in the next section.

### Scoring function

In this section, we describe our scoring function, *f*(*X*,*γ*), which is a linear combination of various terms,

$$\begin{array}{@{}rcl@{}} f(X, \gamma) &=& b(X) + h_{clu-den}(X) + c_{clu-dis} \cdot h_{clu-dis}(X) \\ &&+ c_{clu-size} \cdot \sum_{s=2}^{S_{\max}} h_{clu-size,s}(X,\gamma) + c_{hy} \cdot h_{hy}(\gamma) \\ &&+ c_{pro-num} \cdot h_{pro-num}(X) \end{array} $$

where *S*_max_ is the upper bound on the size of a predicted cluster. The default value is simply set to be 100, and *c*_*clu*−*dis*_ *c*_*clu*−*size*_,*c*_*hy*_, and *c*_*pro*−*num*_, are the coefficients of the corresponding terms.

Here, we briefly explain each term. After that, we give their details. The first term, *b*(*X*), checks the minimum requirements for the predicted cluster of *X*. Whenever there is a cluster in *X* violating at least one of them, the resulting probability of *X* is zero. The second term, *h*_*clu*−*den*_(*X*), calculates the negative of the sum of a generalized density of a predicted cluster in *X*. The effectiveness of these two terms for protein complex prediction is empirically shown in our previous works [4, 12]. The term of *h*_*clu*−*dis*_(*X*) is a newly introduced regularizer to penalize overlaps between predicted clusters of *X*. The remaining terms, $\sum _{s=2}^{S_{\max }} h_{clu-size,s}(X,\gamma), h_{hy}(\gamma)$, and *h*_*pro*−*num*_(*X*), are regularization terms refined from the original ones of the previous works. The group of terms, $\sum _{s=2}^{S_{\max }} h_{clu-size,s}(X,\gamma)$, is a regularizer that checks how the distribution of the sizes of predicted clusters in *X* is similar to the power-law distribution of the scaling exponent *γ*. The term of *h*_*pro*−*num*_(*X*) is also another regularizer that restricts the number of proteins included in *X*.

#### Basic constraints on the model of a protein complex

The Boolean term, *b*(*X*), checks whether every cluster in *X* satisfies basic criteria so that it is reasonable as a predicted cluster. The resulting probability of *X* is set to be zero whenever some of those criteria are false. We require the following two basic constraints on a cluster of proteins, *x*(⊆*V*). One is that the size of *x* should be at most *S*_max_. We simply set the default value of *S*_max_ to be 100. The other constraint is that the vertex-induced subgraph of *G* by *x* should be connected. Namely, every pair of proteins in *x* should have a path via PPIs within *x*. The logical product of the two constraints is represented by the binary function

$${\selectfont{\begin{aligned} {}b(x)\! \,=\,\! \left\{ \begin{array}{ll} \!0 &\! \!\text{if \(|x| \le S_{\max}\)} \\ &\! \!\text{and the vertex-induced subgraph of \textit{G} by \textit{x} is connected,} \\ \!\infty & \!\!\text{otherwise.} \end{array} \right. \end{aligned}}} $$

We then define

$$ b(X) = \sum_{x \in X} b(x). $$

Thus, whenever *X* includes a cluster violating one of the above constraints, the resulting probability density, $P\left (-\frac {b(X)}{T} \right)$, becomes zero, and one otherwise.

The minimum size of predicted clusters is set to be two in our method since a true complex has two or more components. The Boolean term does not include this minimum size requirement because our procedure never produces a predicted cluster with fewer than two components.

#### Density measure

The term *h*_*clu*−*den*_(*X*) evaluates the density of predicted clusters in *X*, in which a generalized density measure for a cluster, *x*⊆*V*,

$$\mathit{density}(x) = \frac{w(x)}{\sqrt{|x|}} $$

is used. The feature of this density measure is that the sum of the weights of all interactions within *x* is divided by $\sqrt {|x|}$ to alleviate excessively severer evaluation of a larger cluster. The standard (weighted) density measure is

$$ \frac{w(x) }{ |x|\cdot (|x|-1)/2 }, $$

the sum of the weights of the interactions within the cluster divided by the possible number of interactions, which is *O*(|*x*|^2^). However, it is not physically reasonable that every pair of proteins within a large complex has an interaction. In this sense, it is not appropriate to use the standard density. Thus, we have reduced the order of the denominator from 2 to 0.5. This density measure was introduced in our previous work [4], and some deeper discussion on the generalized density measure is given in [12]. Based on the density measure for a cluster, *x*, the cost function, *h*_*clu*−*den*_(*X*), over *X* to be minimized is formulated as

$$ h_{clu-den}(X) = - \sum_{x \in X} \mathit{density}(x). $$

#### Regularizing overlaps of clusters

One of the mathematical models representing a set of predicted clusters of proteins is a partition of all proteins of a given set of PPIs, where each element with two or more components in the partition represents a predicted cluster. For example, this model is adopted by MCL [8], SPICi [13], and PPSampler2 [4]. If those clusters could be allowed to slightly overlap with each other, the predictability of those tools is expected to be improved by identifying overlapping complexes. We then design a regularization term that gives a larger penalty for a larger overlap (or say, less dissimilar) between two predicted clusters.

The dissimilarity term between two predicted clusters is formulated based on the Jaccard index as follows. For convenience, we denote by $m_{x,x'}\phantom {\dot {i}\!}$ the minimum size of *x*,*x* ^′^⊆*V*, that is, $\phantom {\dot {i}\!}m_{x,x'} = \min \{ |x|,|x'| \}$. The dissimilarity between *x* and *x* ^′^ is defined as

$${} h_{clu-dis}(x,x') = \left\{ \begin{array}{ll} J(x,x') & \text{if } m_{x,x'} \le 3\ \text{and}\ | x \cap x' | \le 1, \\ & \text{or } m_{x,x'} \ge 4\ \text{and} \frac{| x \cap x' | }{ m_{x,x'}} \le \beta, \\ \infty & \text{otherwise.} \end{array} \right. $$

Namely, we use different criteria for the small clusters with two or three components and for the larger ones. If one of *x* and *x* ^′^ has two or three components, *x* and *x* ^′^ are allowed to share only one protein. This constraint is reasonable given their smallness. If both of *x* and *x* ^′^ have four or more components and the ratio of the number of shared proteins to the minimum number of components is less than or equal to *β*, the penalty is the Jaccard index, *J*(*x*,*x* ^′^), and *∞* otherwise. We then formulate the term *h*_*clu*−*dis*_(*X*) as follows,

$$ h_{clu-dis}(X) = \sum_{x,x' \in X} h_{clu-dis}(x,x'). $$

Note that this dissimilarity measure has a similar role to the repulsive force term used in the task of simultaneously finding multiple sequence motifs [14].

#### Regularizing the distribution of cluster sizes

**Fig. 1:**
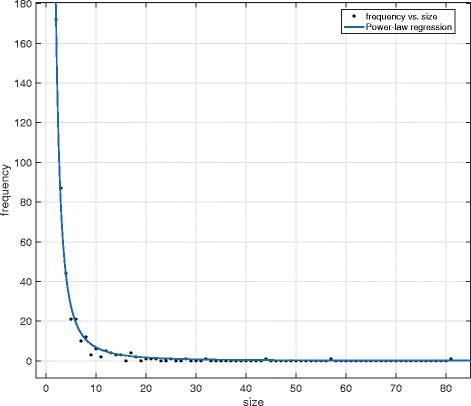
Distribution of protein complex size. The x-axis shows the number of components of protein complexes in CYC2008. The y-axis represents the number of those complexes

The graph in Fig. 1 shows a long-tailed distribution of the sizes of the protein complexes in CYC2008 [6], a yeast protein complex database. The complexes have 2 to 81 components, shown on the *x*-axis. The graph also gives a power-law regression curve, which is proportional to *s* ^−2.02^ with *s*∈[2,100]. Thus, the scaling exponent is 2.02. The root-mean-square error is 1.75. Furthermore, a human protein complex database, CORUM [7], also has the same tendency. Thus, it is reasonable to exploit this power-law feature as prior knowledge to regulate a *set* of predicted clusters.

Thus, we regularize the distribution of the sizes of predicted clusters in *X* by a two-sided truncated power-law distribution over the range [2,*S*_max_]. The probability of cluster size, *s*, in the power-law distribution with a scaling exponent, *γ*, is formulated as

$$\psi_{\gamma}(s) = \frac{1}{ \sum_{t=2}^{S_{\max}} t^{-\gamma}} \cdot s^{-\gamma} $$

where *s*=2,3,…,*S*_max_. We denote by *ψ*_*X*_(*s*) the fraction of predicted clusters with *s* components in *X*, that is,

$$ \psi_{X}(s) = \frac{| \{ x \in X | |x| = s \} | }{|X|}. $$

Then, we define the term *h*_*clu*−*size*,*s*_(*X*) as the square error between *ψ*_*X*_(*s*) and *ψ*_*γ*_(*s*), that is,

$$ h_{clu-size,s}(X) = \left(\psi_{X}(s) - \psi_{\gamma}(s) \right)^{2}. $$

The term *h*_*hy*_(*γ*) is a prior distribution of *γ*, which is defined as a quadratic loss function, that is,

$$ h_{hy}(\gamma) = (\gamma - \gamma_{0})^{2}. $$

The parameter *γ*_0_ is set to be 2.5, the median of the interval, (2,3), which is the typical range of a scaling exponent of power-law distributions in physics, biology, and the social sciences [15]. Note that this prior distribution of *γ* is introduced in this work, although *γ* was fixed to be 2 in the previous work [4], which is almost the same as 2.02, the scaling exponent of the power-law regression curve mentioned above.

#### Regularizing the number of proteins in clusters

Using the term *h*_*pro*−*num*_(*X*), we also control the total number of proteins over all predicted clusters in *X*. The term is simply formulated as the square of that number, that is,

$$h_{pro-num}(X) = \left| \bigcup_{x \in X} x \right|^{2}. $$

This term provides a force to reduce the number of proteins within clusters of *X*. Thus, it can be expected that this term contributes to form more reliable predicted clusters.

This term is simpler than the corresponding term, $\left (\left | \bigcup _{x \in X} x \right | - \lambda \right)^{2}$, given in the previous work [4, 12], where *λ* is a parameter representing a target number of proteins over all clusters. Thus, we do not need to specify that parameter in our new method.

### Proposal function

In general, a proposal function of the Metropolis-Hastings algorithm provides a candidate state of the next iteration that is slightly and randomly modified from the current state. The proposal function used in Algorithm ?? first randomly chooses one of the following four procedures with probabilities, *α*_*a*,*c*_,*α*_*a*,*p*_,*α*_*r*,*c*_, and *α*_*r*,*p*_, respectively (The subscripts of “a”, “r”, “c”, and “p” stand for “addition”, “remove”, “cluster”, and “protein”, respectively):

- randomly add a new cluster with two components to a set of predicted clusters, *X*,
- randomly add a new protein to a cluster in *X*,
- randomly remove a cluster with two components in *X*, and
- randomly remove a protein from a cluster in *X*.

Details of the four procedures are explained in the subsequent sections. After executing one of the above four options, the proposal function subsequently proposes a new candidate value of *γ*, which is max{10^−10^,*γ*+*ε*} where $\varepsilon \sim \mathcal {N}(0,0.001)$. Note that $\mathcal {N}(\mu,\sigma ^{2})$ is the normal distribution with mean parameter, *μ*, and variance parameter, *σ* ^2^. The minimum value of 10^−10^ is used to avoid the value *γ* being negative.

#### Adding a new cluster with two components

In this option, an interaction, *e*∈*E*, is randomly chosen with the probability proportional to the weight, *w*(*e*). Let *x*_*e*_ be the cluster formed with the two proteins of *e*. Then, *x*_*e*_ is added to *X*. As a result, a candidate state *X* ^′^ is given as *X*∪{*x*_*e*_}. The total probability of this proposal, denoted by *Q*_*a*,*c*_(*X* ^′^|*X*), is

$$ Q_{a,c}(X' | X) = \alpha_{a,c} \cdot \frac{w(e)}{\sum_{e' \in E} w(e')}. $$

If the same cluster has already existed in *X*, *X* ^′^ is set to be *X*.

#### Adding a protein to a cluster

For a cluster of proteins, *x*, we denote by *N*(*x*) the set of neighboring proteins to *x*, *i.e.*,

$$ N(x) = \{ u \in V | u \not\in x, \exists v \in x, \{ u, v \} \in E \}. $$

The procedure of adding a protein to a cluster in *X* is as follows:

1. A cluster, *x*, is uniformly chosen at random from *X*.
2. A protein, *u*, is randomly chosen from *N*(*x*) with probability proportional to *w*(*u*,*x*), which is the sum of the weights of the interactions between *u* and all components of *x*.
3. The chosen protein, *u*, is added to *x*.

The resulting state is *X* ^′^. The resulting probability of this proposal is

$$ Q_{a,p}(X' | X) = \alpha_{a,p} \cdot \frac{1}{|X|} \cdot\frac{w(u,x)}{ \sum_{v \in N(x)} w(v,x) }. $$

If *N*(*x*) is empty, *X* ^′^ is the same as *X*.

#### Removing a cluster with two components

This procedure removes a cluster with two components from *X*. It chooses a cluster, *x*, of size two from *X* at random with probability proportional to the inverse of the weight of the unique interaction of *x*. The probability of this proposal is given as

$$Q_{r,c}(X' | X) = \alpha_{r,c} \cdot \frac{ 1/w(x) }{ \sum_{x' \in X \text{ s.t.} |x'| = 2} 1/w(x') }. $$

If such an *x* does not exist, *X* ^′^ is equal to *X*.

#### Removing a protein from a cluster

The last option removes a protein from a cluster by the following procedure.

1. A cluster, *x*, is uniformly chosen at random from the clusters with three or more components in *X*.
2. A protein, *u*, in *x* is randomly chosen with probability proportional to 1/*w*(*u*,*x*), representing the inverse of the strength of the connectivity between *u* and *x*.
3. The chosen protein, *u*, is removed from *x*.

Thus, the resulting probability is

$$ Q_{r,p}(X' | X) = \alpha_{r,p} \cdot\frac{1}{ | \{ x' \in X | |x'| \ge 3 \} |} \cdot \frac{1/w(u,x)}{ \sum_{v \in x} 1/w(v,x) }. $$

If *X* does not include any clusters with three or more components, *X* ^′^ becomes *X*.

### Cooling schedule for the temperature

We denote the value of the temperature parameter of the *ℓ*-th iteration of Algorithm 1 by *T*_*ℓ*_, which is simply formulated as follows. Let *T*_0_ be the initial temperature. It is gradually reduced from *T*_0_(=1) by

$$ T_{\ell} = T_{\ell -1} \times 0.999999. $$

### Performance measure

We use the same performance measure as in [16, 17], which can be described as follows. We say that *x* matches *k* with matching threshold *η* if *J*(*x*,*k*)≥*η*. Let *X* be a set of all clusters predicted by a method, and *K* be a set of all known complexes. For subsets, $\mathcal {X} \subseteq X$ and $\mathcal {K} \subseteq K$, we use the following two sets,

$$\begin{array}{@{}rcl@{}} N_{\mathit{pc}}\left(\mathcal{X}, \mathcal{K}, \eta\right) &=&\{ x | x \in \mathcal{X}, \exists k \in \mathcal{K}, J(x,k) \ge \eta \},\\ N_{\mathit{kc}}\left(\mathcal{X}, \mathcal{K}, \eta\right) &=&\{ k | k \in \mathcal{K}, \exists x \in \mathcal{X}, J(x,k) \ge \eta \}. \end{array} $$

The former represents the subset of $\mathcal {X}$, each of which matches at least one known complex in $\mathcal {K}$ with *η*. The latter is the subset of $\mathcal {K}$, each of which matches at least one predicted cluster in $\mathcal {X}$ with *η*. For an integer *i* (≥2), we denote by *X*|_*i*_ the subset of *X* whose elements have *i* components, that is, *X*|_*i*_={*x*∈*X*||*x*|=*i*}, and by *X*|_≥*i*_ the subset of *X* whose elements have *i* or more components, that is, *X*|_≥*i*_={*x*∈*X*||*x*|≥*i*}. Similarly, we introduce the notations of *K*|_*i*_ and *K*|_≥*i*_ for *K*. We then formulate the *precision* and *recall* as follows:

$$\begin{array}{@{}rcl@{}} &&\mathit{precision}(X, K)\\ &=&\frac{1}{|X|} \cdot\left(| N_{\mathit{pc}}\left(X|_{2}, K|_{2}, 1\right) | +| N_{\mathit{pc}}\left(X|_{3}, K|_{3}, 1\right) | + | \right.\\ &&\quad\left. \times N_{\mathit{pc}}\left(X|_{\ge 4}, K|_{\ge 4}, 0.5\right)|\right),\mathit{recall}(X, K)\\ &=&\frac{1}{|K|} \cdot\left(| N_{\mathit{kc}}\left(X|_{2}, K|_{2}, 1\right) | +| N_{\mathit{kc}}\left(X|_{3}, K|_{3}, 1\right) | +| \right.\\ && \quad\left. \times N_{\mathit{kc}}\left(X|_{\ge 4}, K|_{\ge 4}, 0.5\right) | \right). \end{array} $$

Notice that the matching threshold for predicted clusters and known complexes with four or more components is set to be *η*=0.5. On the other hand, the matching criterion for predicted clusters and known complexes with two or three components is an exact match as *η*=1. The reason for this is as follows. In many works on the problem of protein complex prediction, the degree of overlap between a predicted cluster, *x*, and a known complex, *x* ^′^, is measured by the Jaccard index, $J(x,x') = \frac {|x \cap x'| }{ |x \cup x'| }$, or the ratio of the size of the intersection between *x* and *x* ^′^ to the geometric mean of |*x*| and |*x* ^′^|, that is, $\frac {|x \cap x'| }{ \sqrt {|x|\cdot |x'|} }$. These measures do not work well for small sizes if a threshold is low. For example, consider the case where *x* and *x* ^′^ with |*x*|=|*x* ^′^|=2 share exactly one protein. Note that this situation is easily realized by randomly predicting clusters with two components because there are many known complexes with two components in protein complex datasets. In this case, we see that *J*(*x*,*x* ^′^)=1/3 and the other ratio is 1/2. Thus, *x* and *x* ^′^ are determined to match with each other by both measures if the threshold is set to be less than or equal to 1/3. We avoid this issue by setting the threshold to be one for small clusters and complexes. The *F-measure* of *X* to *K* is the harmonic mean of the corresponding precision and recall, that is,

$$\mathit{F}(X, K) = 2 \cdot \frac{\mathit{precision}(X, K)\cdot\mathit{recall}(X, K)}{\mathit{precision}(X, K)+\mathit{recall}(X, K)}. $$

## Results and discussion

### Input PPI datasets and gold standard protein complexes

A set of PPIs with weights is given as input to a protein complex prediction method. Our main PPI dataset is the WI-PHI database [18]. Every PPI of the dataset is assigned a weight representing its reliability derived from various heterogeneous data sources. Any PPI of the dataset except self-loop interactions is not filtered out by a threshold to the weight. The number of proteins is 5953 and that of non-self-loop PPIs is 49,607, as shown in Table 1. On average, a protein has 16.7 interactions with others. The weights of the PPIs range from 6.6 to 146.6. The normalized weights, which are divided by the maximum value, are given to protein complex prediction methods.

**Table 1:** Input PPI datasets

	#Protein	#PPI	Degree	Threshold
WI-PHI	5,953	49,607	16.7	N/A
Collins	1,622	9,074	11.2	top 9,074
Krogan core	2,708	7,123	5.3	0.273
Krogan extended	3,672	14,317	7.8	0.101
Gavin	1,855	7,669	8.3	5

This table shows the number of proteins, the number of PPIs, the average of the degrees of proteins, and the threshold used to filter out unreliable PPIs

In addition to the WI-PHI dataset, we also use four different datasets of PPIs with weights, which are denoted by Collins [19], Gavin [20], Krogan core, and Krogan extended [21], which were also used in [9]. As shown in Table 1, the number of proteins included in each dataset is much smaller than the number of all yeast proteins, which is about 6,000. Those datasets are filtered by the threshold of those weights, shown in Table 1, to use reliable PPIs. Those thresholds are the same as in the original papers [19–21] of the PPI datasets and the work [9].

All protein complexes in the yeast protein complex database, CYC2008 [6], are used as gold standard protein complexes. As mentioned before, an interesting point is that among the complexes, 172 (42%) and 87 (21%) complexes have two and three components, respectively. It has 216 pairs of two complexes overlapping with each other, and those pairs are formed with 112 complexes. Details are given in Table 2.

**Table 2:** The frequency of overlap sizes of protein complexes in CYC2008

Overlap size	1	2	3	4	5	6	7	8	9	10	11	12	13	14	15	16	17
Frequency	151	22	9	13	4	1	10	1	0	1	1	1	0	0	0	1	1

The row of “Overlap size” shows the size of the intersection between two complexes. The row of “Frequency” gives the number of overlapping complexes

### Performance comparison

To evaluate how RocSampler works well, we carry out a performance comparison with existing methods, MCL [8], SPICi [13], ClusterONE [9], NWE [11], and PPSampler2 [4]. For each tool and each PPI dataset, the parameter set with the highest F-measure is determined as follows. MCL is a popular clustering-based method. It alternately repeats two different steps. One is the expansion step, which takes the square of a current transition matrix of an input PPI network. Another is the inflation step, in which all transition probabilities are raised to the power of the value of the inflation parameter and normalized. The inflation parameter is optimized over the range from 1.2 to 5.0 in steps of 0.1. SPICi is a clustering algorithm using the weighted version of the standard density measure. The parameters of minimum cluster density and minimum support threshold are independently chosen in the range from 0.1 to 0.9 in steps of 0.1. The graph mode parameter is also optimized over 0 (sparse graph), 1 (dense graph), and 2 (large sparse graph). ClusterONE is also a clustering algorithm using a cohesiveness score. The most important parameter is the minimum density of predicted complexes. We optimized the parameter value over the range from 0.1 to 0.9 in steps of 0.1. NWE executes random walks with restarts and constructs predicted clusters based on the probability from one protein to another obtained from the random walks. Here, three parameters are optimized. The restart probability takes the range from 0.4 to 0.8 in steps of 0.1. The early cutoff is optimized in the range from 0.3 to 0.7 in steps of 0.1. The overlap threshold is selected from the range from 0.1 to 0.4 in steps of 0.1. PPSampler2 is an MCMC(Markov Chain Monte Carlo)-based method whose structure of a set of predicted clusters is a partition of proteins. The following four parameters are optimized. The coefficient of the term regulating the power-law distribution of sizes of predicted clusters is selected among 500, 1000, and 1,500. The scaling exponent is optimized over 2.0, 2.5, and 3.0. The coefficient of the term regulating the number of proteins over predicted clusters is selected from 10^5^, 10^6^, and 10^7^. The target number of proteins used in that term, *λ*, is selected from 1,000, 2,000, and 3,000. The four coefficients of the scoring function of RocSampler are optimized over the ranges: *β*∈{0.2,0.3,0.4}, *c*_*clu*−*size*_∈{200,300,400,500}, *c*_*hy*_∈{5,10,15,20}, *c*_*pro*−*num*_∈{5×10^−5^,10^−4^,1.5×10^−4^}, and *c*_*clu*−*dis*_∈{70,90,110,130,150,170}. The repeat count, *L*, is fixed to 5,000,000.

Note that MCL, SPICi, and PPSampler2 do not allow predicted clusters to overlap with each other.

#### Prediction from WI-PHI

**Fig. 2:**
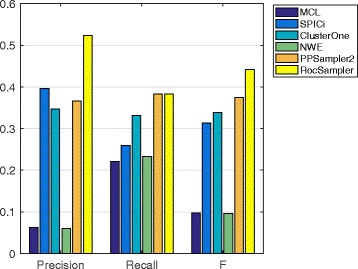
Precision, recall, and F-measure of MCL, SPICi, ClusterONE, NWE, PPSampler2, and RocSampler on the WI-PHI PPI dataset

The selected parameter values on the WI-PHI PPI dataset are shown in Table 3, and the precision, recall, and F-measure are given in Fig. 2. Regarding precision, the methods are classified into three groups. The top group comprises only RocSampler, which achieved a remarkably high precision score, 0.52. This score is derived from 147 correctly predicted clusters out of 281 predicted ones. The second group consists of SPICi, PPSampler2, and ClusterONE, whose scores are 0.40, 0.37, and 0.35, respectively. The third group consists of the remaining tools, MCL and NWE, whose scores are drastically low, at about 0.06. Regarding recall, RocSampler and PPSampler2 obtain the same highest score, 0.38. This score is obtained from 156 predicted clusters matched with at least one known complex over all 408 known complexes. The third best score, 0.33, is achieved by ClusterONE. The scores of the remaining tools are less than 0.26. Recall that F-measure is the harmonic mean of precision and recall. Regarding this measure, RocSampler clearly outperforms the other tools. The F-measure score is 0.44, followed by 0.37 (PPSampler2), 0.34 (ClusterONE), and 0.31 (SPICi).

**Table 3:** Selected parameters

	Parameters	Value
MCL	Inflation	3.4
SPICi	Density, support, graph	0.1, 0.5, 0
ClusterONE	Density	0.2
NWE	Restart, cutoff, overlap	0.4, 0.3, 0.3
PPSampler2	Size dist coef, scaling exp,	500, 3,
	Protein num coef, *λ*	10^6^, 2000
RocSampler	*c*_*clu*−*dis*_, *β*, *c*_*clu*−*size*_,*c*_*hy*_,	110, 0.2, 500, 10,
	*c*_*pro*−*num*_,	5×10^−5^

We here compare the performances of PPSampler2 and RocSampler intensively, because, RocSampler is an improved version of PPSampler2. The precision scores of PPSampler2 and RocSampler are 145/396 = 0.37 and 147/281 = 0.52, respectively. On the other hand, their recall scores are, as mentioned, the same, 156/408 = 0.38. Thus, RocSampler improves the precision score without reducing the recall score. As a result, the F-measure score of RocSampler, 0.44, is 19% higher than that of PPSampler2, 0.37.

**Fig. 3:**
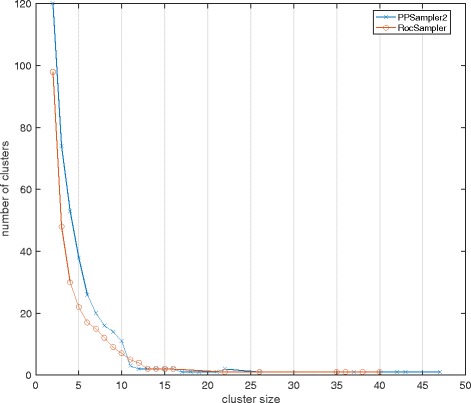
The distributions of sizes of predicted clusters by PPSampler2 and RocSampler

We furthermore compare details of the predictions by PPSampler2 and RocSampler. Figure 3 shows the distributions of the sizes of predicted clusters of PPSampler2 and RocSampler. We can see that PPSampler2 predicted more clusters with two to ten components. These extra clusters just make the precision score of PPSampler2 worse than that of RocSampler because both of the recall scores are the same.

Surprisingly, no predicted clusters of RocSampler overlap with others, although we had expected that some would overlap with each other. A relatively sparse set of predicted clusters might be a good approximation to the current gold standard protein complexes, although further investigation of this issue is required.

We have mentioned that the scaling exponent of the power-law regression curve in Fig. 1 is 2.02. The found value of *γ* is 1.91, which is quite similar to the true value.

#### Prediction from other PPI datasets

**Fig. 4:**
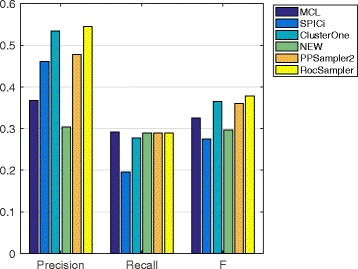
Prediction performance on the Collins PPI dataset

**Fig. 5:**
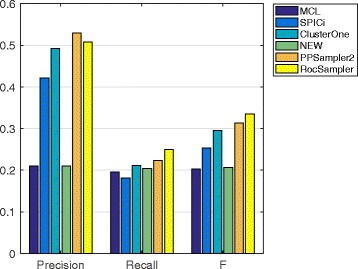
Prediction performance on the Gavin PPI dataset

**Fig. 6:**
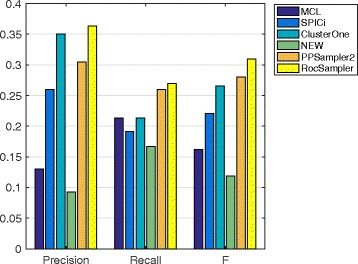
Prediction performance on the Krogan core PPI dataset

**Fig. 7:**
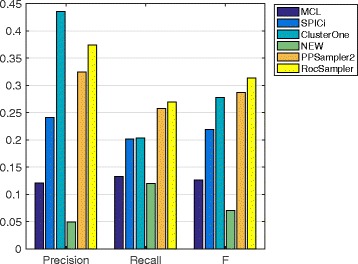
Prediction performance on the Krogan extended PPI dataset

The prediction performances of the methods on the four remaining PPI datasets are given in Figs. 4, 5, 6 and 7. The chosen best parameter values are given in Table 4. As we can see, RocSampler is superior to the other methods in F-measure for each PPI dataset. In addition, RocSampler also outperforms the others at least in either precision or recall.

**Table 4:** Selected parameters for the Collins, Gavin, Krogan core, and Krogan extended PPI datasets

	Collins	Gavin	Krogan core	Krogan extended
MCL	2.1	2.5	2.4	1.6
SPICi	0.1, 0.5, 0	0.4, 0.4, 0	0.6, 0.3, 0	0.6, 0.4, 0
ClusterONE	0.7	0.4	0.6	0.7
NWE	0.4, 0.3, 0.1	0.4, 0.3, 0.2	0.4, 0.3, 0.4	0.4, 0.7, 0.1
PPSampler2	1500, 3, 10^7^, 1000	500, 2.5, 10^5^, 1000	1000, 2, 10^7^, 1000	1500, 3, 10^7^, 1000
RocSampler	90, 0.3 300, 5,	170, 0.2, 200, 20,	170, 0.3, 500, 15,	150, 0.3, 200, 15,
	1.5×10^−4^	10^−4^	1.5×10^−4^	1.5×10^−4^

#### Example of overlapping clusters

RocSampler has succeeded in predicting overlapping clusters only from the Collins PPI dataset. We here give an example of such overlapping clusters, which are good predictions of known complexes.

**Fig. 8:**
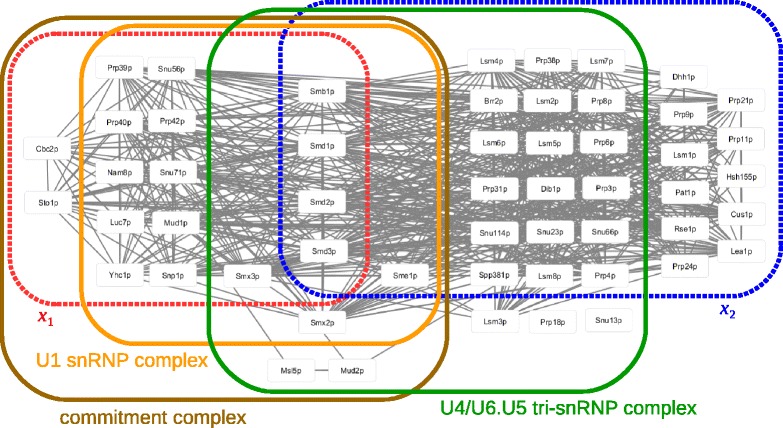
Example of overlapping clusters. The red and blue broken lines surround the proteins included by two predicted clusters. The brown, orange, and green lines surround the proteins included by the known complexes that match one of the predicted clusters

Figure 8 shows two overlapping clusters and their matched known complexes. The clusters are represented by red and blue broken curves, denoted by *x*_1_ and *x*_2_, which surround their component proteins. As we can see, they share the four proteins, Smb1p, Smd1p, Smd2p, and Smd3p. These four proteins are known to be part of the heteroheptameric complex with Sme1p, Smx3p, and Smx2p, which are also shown in Fig. 8. The heteroheptameric complex is known as part of the spliceosomal U1, U2, U4, and U5 snRNPs. snRNPs (small nuclear ribonucleo proteins), which are RNA-protein complexes, form a spliceosome with unmodified pre-mRNA and various other proteins. Thus, it can be expected that *x*_1_ and *x*_2_ match some of the spliceosomal snRNPs. Actually, as shown in Fig. 8, *x*_1_ matches the U1 snRNP complex [22] with Jaccard index 0.79, whose components are surrounded by an orange solid curve. In addition, *x*_1_ overlaps more with the commitment complex with Jaccard index 0.81, indicated by a brown solid curve. The commitment complex is known as an ATP-independent complex that commits hnRNAs to the splicing pathway [23]. Furthermore, *x*_2_ matches the U4/U6.U5 tri-snRNP complex [24, 25] whose Jaccard index is 0.59, indicated by a green solid curve in Fig. 8.

On the other hand, PPSampler2 found the cluster with Mud1p, Luc7p, Prp42p, Snu56p, Snu71p, Nam8p, Snp1p, Prp40p, Yhc1p, Prp39p, Sto1p, Cbc2p, and Smx3p. This cluster includes only Smx3p among the seven components of the heteroheptameric complex. Although it matches the commitment complex and U1 snRNP complex, the Jaccard indexes are 0.61 and 0.58, lower than the corresponding ones of RocSampler. It can be expected that all or most of the remaining components of the heteroheptameric complex are included in another cluster which matches the U4/U6.U5 tri-snRNP complex, but PPSampler2 failed to find such a cluster. Thus, we can say that, by allowing predicted clusters to overlap with each other, more refined predictions are obtained.

## Conclusion

In this work, we have proposed a novel sampling-based protein complex prediction method, RocSampler, which is a successor to PPSampler2. The major difference between them is that RocSampler exploits a regularization term for controlling overlaps of predicted clusters and PPSampler2 does not allow predicted clusters to overlap with each other. RocSampler also introduced a new proposal function for generating overlapping clusters and regularization terms refined from those of PPSampler2. We have shown that RocSampler outperforms five other methods on five different PPI datasets. RocSampler has succeeded in finding overlapping clusters from the Collins PPI dataset, but it has not done so from the other PPI datasets. Future work is required to identify the reason for this and to devise a new scoring function to attain higher performance and simultaneously to find overlapping clusters of proteins.
